# A Novel Intronic Splice—Site Mutation of the *CYP11A1* Gene Linked to Adrenal Insufficiency with 46,XY Disorder of Sex Development

**DOI:** 10.3390/ijerph18137186

**Published:** 2021-07-05

**Authors:** Pawel Matusik, Agnieszka Gach, Olimpia Zajdel-Cwynar, Iwona Pinkier, Grzegorz Kudela, Aneta Gawlik

**Affiliations:** 1 Department of Pediatrics and Pediatric Endocrinology, Faculty of Medical Sciences in Katowice, Medical University of Silesia, 40-752 Katowice, Poland; endo_sk6@sum.edu.pl; 2 Department of Genetics, Polish Mother’s Memorial Hospital Research Institute, 93-338 Lodz, Poland; agagach@tlen.pl (A.G.); iwona.pin@interia.pl (I.P.); 3 Upper-Silesian Child’s Health Centre, 40-752 Katowice, Poland; olimpiacwynar@gmail.com; 4 Department of Pediatric Surgery and Urology, Faculty of Medical Sciences in Katowice, Medical University of Silesia, 40-752 Katowice, Poland; gkudela@sum.edu.pl

**Keywords:** *CYP11A1* gene, disorder of sex development (DSD), congenital adrenal hyperplasia (CAH), novel mutation

## Abstract

A novel *CYP11A1*: c.1236 + 5G > A was identified, expanding the mutation spectrum of the congenital adrenal insufficiency with 46,XY sex reversal. In a now 17-year-old girl delivered full-term (G2P2, parents unrelated), adrenal failure was diagnosed in the first year of life based on clinical picture of acute adrenal crisis with vomiting, dehydration, weight loss, hypotension, and electrolyte disturbances. At the time, hormonal tests revealed primary adrenocortical insufficiency and steroid profiles showed lack of products of steroidogenesis, and since then the patient has been treated with substitution doses of hydrocortisone and fludrocortisone. At the age of 14, considering the absence of puberty symptoms, extended diagnostic tests revealed elevated LH levels (26.5 mIU/mL) with pre-puberty FSH levels (4.9 mIU/mL), low estradiol (28 pmol/L), testosterone (<2.5 ng/mL), and extremely high levels of ACTH (4961 pg/mL). A cytogenetic study revealed a 46 XY karyotype. A molecular examination confirmed the missense mutation and a novel splice-site mutation of *CYP11A1* gene. Compound heterozygosity for the *CYP11A1* gene with a known pathogenic variant in one allele and a novel splice site mutation in the second allele is most probably responsible for congenital adrenal insufficiency with 46,XY sex reversal. We discuss the necessity of cytogenetic test in the case of early onset of adrenal failure in the absence of steroidogenesis metabolites in the steroid profile.

## 1. Introduction

**Figure 1:**
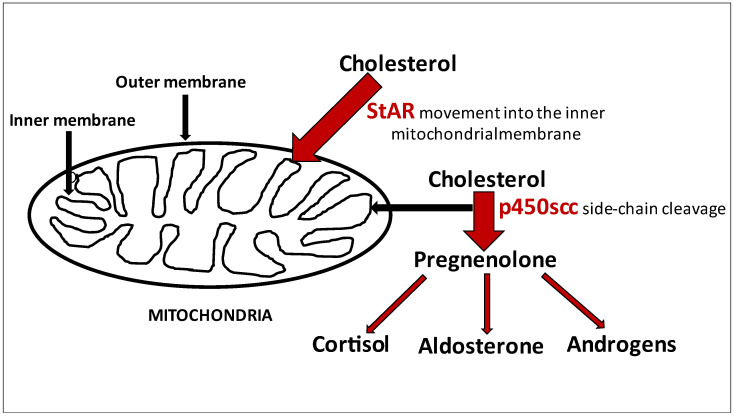
Two initial steps of steroidogenesis related to the StAR and p450scc activity.

Congenital adrenal hyperplasia (CAH) is a rare autosomal recessive disease caused by mutations of genes for enzymes that are essential for steroidogenesis. The most severe form of CAH, characterized by the defect in both adrenal and sex steroids synthesis, is caused by mutation in the steroidogenic acute regulatory (StAR) protein and mutation in the cholesterol side-chain cleavage enzyme gene *CYP11A1*. The defect in StAR causes lipoid congenital adrenal hyperplasia (LCAH) with the characteristic massively enlarged adrenals filled with lipids. Due to the deficiency of StAR protein, the transport of cholesterol to the mitochondria is impaired and the conversion of cholesterol to pregnenolone is reduced. Consequently, steroidogenesis is impaired [1]. However, the first rate-limiting step in the biosynthesis of steroid hormones is the conversion of cholesterol to pregnenolone catalyzed by p450-mediated cholesterol side-chain cleavage enzyme (P450scc) coded by the *CYP11A* gene. The highest P450scc activity is present in the adrenal cortex, corpus luteum, ovarian theca cells, and Leydig cells [2]. As pregnenolone is the precursor to all steroid hormones, a homozygous *CYP11A* gene mutation leads to the lack of p450scc leading similarly to StAR deficiency, impaired production of all steroid hormones. The role of both StAR and p450scc in the first steps of steroidogenesis was presented in the Figure 1. Symptoms for both StAR and p450scc mutation include life-threatening adrenal insufficiency in early infancy and female phenotype regardless of their sex chromosome complement. In our study, we present a case of a patient with 46,XY disorder of sex development (DSD) harboring one known missense mutation and one novel intronic splice-site mutation of the *CYP11A1* gene, with the primary diagnosis of Addison’s disease that was admitted to our department to evaluate the disease control and possible modification of the treatment.

## 2. Case Presentation

**Figure 2:**
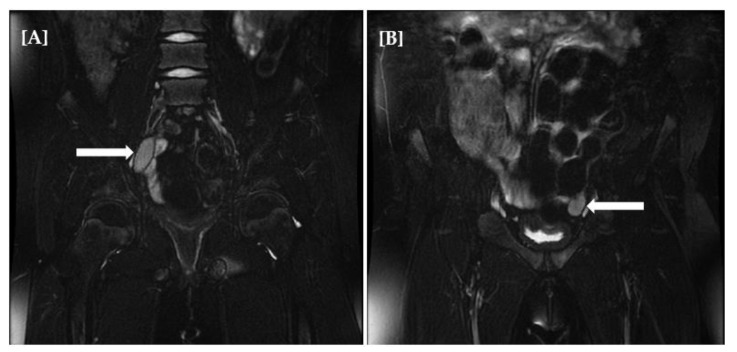
Magnetic resonance imaging (MRI) of abdomen and pelvis minor showing presence of gonads (right (**A**), left (**B**)) with the absence of the structures derived from the Müllerian ducts.

Currently, a 17-year-old girl delivered full-term (G2P2, parents unrelated) with normal birth parameters was diagnosed with adrenal failure in the first year of life. Family history of autoimmune diseases was negative. Hyperpigmentation of the skin was reported when she was ~1 month old. At the age of 10 months, the child had an acute adrenal crisis with a classic symptomatology (vomiting, dehydration, weight loss, hypotension, and electrolyte disturbances). Hormonal tests revealed primary adrenocortical insufficiency, and steroid profiles showed lack of products of steroidogenesis. Classical replacement therapy with hydrocortisone and fludrocortisone has been implemented. In January 2014, the girl was admitted to the clinic to assess the effectiveness of the current doses of medication used. Physical examination demonstrated skin hyperpigmentation, obesity, puberty Tanner I, and female external genitalia. In the absence of puberty symptoms, elevated LH level (26.5 mIU/mL) with either pre-pubertal FSH and estradiol level (4.9 mIU/mL and 28 pmol/L, respectively) was a warning. Moreover, undetectable or very low levels of testosterone (<2.5 ng/mL), DHEAS (<15 µg/dL), and 17-OH progesterone (0.59 ng/mL) were noted. In the repeated steroid profile, apart from metabolites derived from substitution therapy, no other steroidogenesis markers were found. Despite adequate hydrocortisone supply, ACTH levels (4961 pg/mL) were still very high. However, before the admission to the our department, the patient was treated for 11 years in the different center. Magnetic resonance imaging (MRI) of hypophysis did not reveal either hypophysis enlargement or adenoma. In the pelvic ultrasound, the uterus and ovaries were not visualized. A cytogenetic study, by karyotyping with routine G-banding according to the recommendations of the American College of Medical Genetics, revealed a 46,XY karyotype. Magnetic resonance imaging (MRI) confirmed the presence of gonads in the abdomen and the absence of the structures derived from the Müllerian ducts (Figure 2A,B). Based on the clinical picture and the results of additional tests, the *STAR* or *CYP11A1* gene mutation with disorder of sex determination 46,XY karyotype gonadal dysgenesis with adrenal failure was taken into account. In June 2014, the patient was subjected to bilateral laparoscopic gonadectomy. Histopathological examination confirmed a typical testicular tissue with neither gonadal dysgenesis nor lipid storage features. This finding explains the discrepancy in the levels of gonadotrophins in our patient described above. High LH level was related to the lack of testosterone production with no negative feedback and normal FSH level was related to the preserved Sertoli cells function and normal production of inhibin b. A molecular examination was performed to confirm the diagnosis—two variants in the cholesterol side-chain cleavage enzyme gene *CYP11A* were identified. Patient, based on the psychological tests and consultation presents female gender identity and behavior and in follow-up care, estrogen replacement therapy was implemented in July 2014 with continuation of hydrocortisone and fludrocortisone supplementation.

## 3. Methods

DNA was automatically extracted from peripheral blood leukocytes using MagCore Genomic DNA Whole Blood Kit (RBC Bioscience, New Taipei City, Taiwan), according to the manufacturer’s instructions.

Customized panel includes 108 genes and candidate genes associated with DSD (Disorders/Differences of Sex Development). Gene selection was made based on scientific literature and Online Mendelian Inheritance in Man (OMIM) database. The following genes are covered in the panel: *AKAP2, AKR1C2, AKR1C4, AMH (MIS), AMHR2 (MISRII), AR, ARX, ATF3, ATRX, BMP15, BMP4, BMP7, BNC2, CAMK1D, CBX2 (M33), CDKN1C, CITED2, CTNNB1, CUL4B, CYB5A, CYP1A1, CYP11A1 CYP11B1 CYP17A1 CYP19A1, CYR61, DAZL, DGKK, DHCR7, DHH, DKK1, DMRT1, DMRT2, DNAJC15, DPPA3, EBF2, EFEMP1, EGF, EMX2, ESR1, ESR2, EXO1, FGF9, FGF2, FGFR2, FOXL2, FSHR, FZD1, GATA4, GSTM1, GSTT1, HDAC8, HESX1, HHAT, HOXA4, HOXA13, HOXB6, HSD3B1, HSD17B3, HSD17B4, HSD3B2, IGF1R, IFITM1, INSL3, INSR, KANK1, LHCGR, LHFPL5, LHX1 (LIM1), LHX9, MAMLD1, MAP3K1, MCM9, MID1, MTM1, NLGN4X, NMT2, NR0B1, NR5A1, NR3C1, PAX2, PAX8, PBX1, POR, PSMC3IP, PTGDS, RSPO1, SOX3, SOX10, SOX8, SOX9, SPRY2, SRD5A2, SRY, STAG3, STAR, TBX2, TDRD7, TEX10, TSNAX, TSPYL1, TSPYL4, VNN1, WNT4, WT1, WTAP, WWOX, ZFPM2 (FOG2)*.

The probes for the desired targeted regions were designed using the Illumina Design Studio the web-based software providing the 99% sequencing coverage of 1502 amplicons with an average length of 250 bp (2 × 150 base pairs reads length in paired-end mode) for the MiniSeq sequencer (Illumina, Inc., San Diego, CA, USA). Libraries were prepared using TruSeq Custom Amplicon Low Input Library Prep Kit according to the manufacturer’s protocol (Illumina, Inc., San Diego, CA, USA). All DNA samples were quantified and diluted to the concentration of 10 ng/μL. After hybridization, extension and ligation of oligos specific to the targeted regions of interest, the libraries were barcoded, amplified, finally normalized, pooled, and loaded into the cartridge (Illumina MiniSeq High Output Kit, 300 cycles). The PhiX library was combined with prepared library as a sequencing control and submitted to the Illumina MiniSeq platform. Sequencing was performed on the flow-cell using 2 × 150 bp paired-end runs. Analysis was performed using Variant Studio 3.0 software (Illumina), according to human reference genome GRCh37/hg19.

Variants were verified with Sanger sequencing method on an ABI3500 Genetic Analyzer using primers designed with Primer3 Input and Primer-BLAST software.

## 4. Results

**Figure 3:**
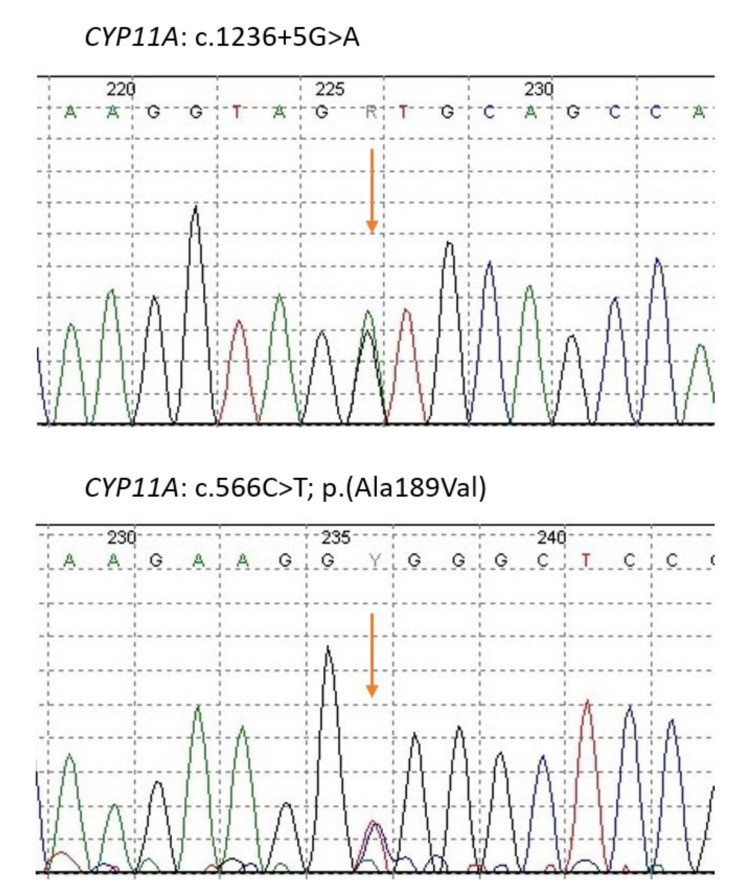
Results of automated DNA sequencing for *CYP11A1* splice site mutation NM_000781.2 c.1236 + 5G > A (**upper** chromatogram) and missense mutation NM_000781.2 c.566C > T; p.(Ala189Val) (**bottom** chromatogram).

Two variants in the cholesterol side-chain cleavage enzyme gene *CYP11A* were identified with custom panel NGS and confirmed with Sanger sequencing (Figure 3). A novel intronic splice-site mutation NM_000781.2 c.1236 + 5G > A was found to be of paternal origin. The variant was predicted to be deleterious by in silico prediction tools: GERP and scSNV-splicing. The Genome Aggregation Database (gnomAD), ExAC, and1000 Genomes Project show no records for the variant’s population frequency.

The second identified variant NM_000781.2 c.566C > T; p.(Ala189Val) is a missense mutation leading to amino acid substitution and was maternally inherited. The variant was previously reported in congenital adrenal insufficiency [3] and deposited in ClinVar (SCV000039357). According to ACMG-AMP recommendations, both variants were classified as class 3, VUS.

**Figure 4:**
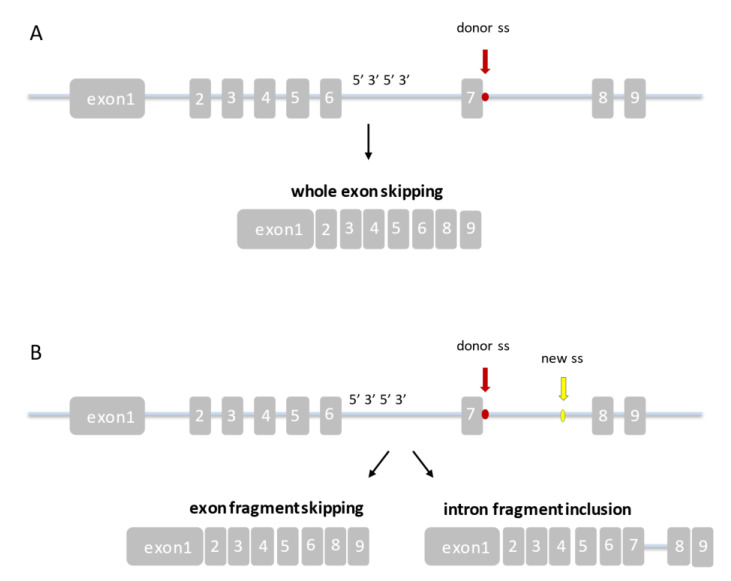
Expected *CYP11A1*: c.1236 + 5G > A mutation effect on splicing. (**A**) Splicing mutation within the canonical splice sites leading to whole exon skipping; (**B**) splicing mutation resulting in the usage of the cryptic exonic or intronic splice site that leads to the inclusion of an intron fragment or exon fragment skipping.

Functional testing for identified novel variant *CYP11A1*: c.1236 + 5G > A was not performed. The point mutation occurs at a donor splice site and is expected to cause a change in the final protein product. This type of variant in canonical splice site can lead to whole exon skipping, or by cryptic splice site activation may cause the inclusion of an intron fragment or exon fragment skipping (Figure 4).

## 5. Discussion

We have identified a novel splice site mutation of *CYP11A1* gene linked to the congenital adrenal insufficiency. In general, mutations in the canonical acceptor and donor sites affect strongly conserved sequences that define exon–intron boundaries. They lead to aberrant pre-mRNA splicing, improper intron removal, and alterations of the open reading frame [4]. As *CYP11A* gene expression is restricted to adrenal glands, gonads, and placenta, it was impossible to perform functional studies. The novel *CYP11A* splice site mutation was predicted to be deleterious by dedicated bioinformatical tools. Similarly, the second identified variant, which was previously reported as pathogenic in a patient with CAH lacks the functional studies, but the in vitro expression analyses showed that the c.666C > T p.(Ala189Val) mutation may introduce an alternative splicing site that partially inactivates the activity of the *CYP11A1* gene [3].

Patients with *CYP11A1* deficiency cannot produce any steroids. The lack of fetal testosterone with a normal anti-Müllerian hormone cause leads to 46,XY genetically male fetuses with female-appearing external genitalia, despite having male internal reproductive structures. The first symptoms can occur soon after birth [5,6] as the most severe forms and patients present signs of adrenal crisis, including electrolyte abnormalities, severe weakness, recurrent vomiting, and seizures. Glucocorticoid deficiency may also cause poor glycemic control and poor stress response. Absence of mineralocorticoids is responsible for hyponatremia, hyperkalemia, and acidosis with accompanying dehydration and death within a first months of life if not diagnosed and treated. The milder forms with delayed onset of adrenal insufficiency manifest in later infancy or childhood [3,7,8,9,10].

In our patient, the *STAR* gene mutation was first considered. We found some similar features to patients with lipoid congenital adrenal hyperplasia. The time of first symptoms of our patient was about 10 months, which has been described in previous studies in patients with proven LCAH [11,12]. Additionally, skin hyperpigmentation was also one of the characteristic symptoms for LCAH patients. Moreover, StAR deficiency would also explain disorder/differences of sex development. We did not find adrenal enlargement in computed tomography, which was proven in the most cases of LCAH. On the other hand, the lack of radiologically adrenal hyperplasia does not exclude the diagnosis of LCAH [13]. The final cause of disease was confirmed using custom panel NGS and Sanger sequencing. Molecular examination revealed two variants in the cholesterol side-chain cleavage enzyme gene *CYP11A*. For the first time, adrenal insufficiency with 46,XY sex reversal was identified in 2001 [10]. One of the mutations we have shown in our study was first reported in a study of a child with adrenal insufficiency features including hyperpigmentation and markedly elevated ACTH levels. In contrast to our patient, the karyotype was 46,XX with normal levels of cortisol and 17-OH progesterone, as well as electrolyte and glucose levels. The compound heterozygosity missense mutation in the *CYP11A1* gene was then identified [3]. This variant in our study is maternally inherited. In the following years, many different types of mutations in the *CP11A1* gene have been described [6,14]. Some of the mutations cause a complete loss of enzyme function, while others retain partial activity, which translates into a spectrum of symptoms [6]. A milder “nonclassical form” of P450scc deficiency caused by missense mutations that retain 10–20% of normal activity has been described recently. A common feature of all variants of p450scc deficiency is the absence of adrenal enlargement typical of lipoid CAH [9].

The latest years have resulted in the discovery of new unknown mutations causing adrenal insufficiency [15,16], which extend the list of genetic variants for CAH. Our study also brought a so far undiscovered mutation of paternal origin, and it is a novel intronic splice-site mutation. Identifying the etiology of adrenal insufficiency presenting during childhood is essential and appropriate steroid hormone replacement therapy leads to long-term survival.

Despite the full compatibility of the clinical picture with the molecular analysis in our patient, we are aware that the main limitation of our study is the lack of a functional analysis of the splicing variant *CYP11A* we found.

## 6. Conclusions

Targeted panel sequencing revealed compound heterozygous variants of the *CYP11A1* gene. A novel *CYP11A1*: c.1236 + 5G > A was identified, expanding the mutation spectrum of the congenital adrenal insufficiency with 46,XY sex reversal.

In case of early onset of adrenal failure in the absence of steroidogenesis metabolites in the steroid profile, LCAH should be considered in differential diagnosis. In addition, in female phenotype children with early onset of adrenal insufficiency, a karyotype is necessary.

## Data Availability

The datasets used and/or analysed during the current study are available from the corresponding author on reasonable request.
